# The Great Plague

**Published:** 1922-01

**Authors:** A. T. Nankivell

**Affiliations:** (Medical Officer of Health, Hornsey)


### NOTABLE EPIDEMICS.

### III.—THE GREAT PLAGUE.

By A. T. NANKIVELL, M.D., D.P.H. (Medical Officer of Health, Hornsey).

THERE was a certain apothecary, William Boghurst by name, who was living in London in the year 1664. He was a just and upright man, and, since he did not flee from the city during those days of pestilence, but remained instead to write an excellent account of it, we learn from him almost all that we know with accuracy regarding the Great Plague of 1664-1666, which ravaged especially the metropolis. It is said that of all the doctors in London only six remained to do their duty to the sick and dying, and that the great Sydenham was among those who fled from the city. Boghurst has little use for the timorous or for their evidence. " The great doctors," says he, " the great doctors and such as undertake to write about the disease are the first that ran away from it, and soe it followes therefore all their learning about it can bee but opiniative and conjecturall." And, again, in his " Preface to the Reader," " I have writ nothing from heresay or from bodies or from the testimony of others or of my own conceit, but all are onely from experience and triall; and I had the more advantage to doe this, being not at all fearfull, but rendering myself familiar to this disease as to any other, whereas many because of their fearfullnesse came not to close practice, but stood shivering at a distance, and profited the less both to themselves and their patients, and commonly lost their lives to boot." The apothecary was a stylist with a pleasant sense of humour.

Daniel Defoe, who was a boy of four years old at the time of the Great Plague, heard, no doubt, many stories of its terrors, for in 1722 he wrote a journal of the Plague year which is certainly not altogether imaginative. In the year 1720 there was a larger outbreak of Plague at Marseilles, and the literature regarding this may have helped to stir the memory of Defoe. His account, which lacks the medical details of that by Boghurst, is, however, an admirable and readable source of information.

There had been sporadic cases of Plague in London for many years; indeed, the disease had hardly been absent since the days of the Black Death. But the incidence and the mortality of these sporadic cases was light. The infection seemed to be attenuated in virulence. Towards the end of December, in 1664, a death from Plague occurred in Long Acre, and the infection in this case was attributed to a bale of silks that had arrived at the house from the Levant. It is certainly possible that a new and virulent strain of plague was introduced into England in this manner; but this explanation is not the most likely, since it was not until the beginning of the following June that the disease became really epidemic. If the suggestion of the importation of plague from abroad be discarded, we must suppose that some factor or factors at home intensified the virulence of our ever-present endemic infection. Boghurst thinks of the Plague that " it must be a poyson created *de novo* for the punishment of sinne "; and of the manner of this creation he writes : " For my owne part, farr bee it from mee to inform punctually concerning the certaine cause of a Pestilence, as noe man hath done, but I think I may have liberty to putt in my opinion also into the number, and I have alwaies had a conceite that seeing tyme produceth and perfiteth all things that the Plague may be generated thus, that in some compasse of yeares or tyme the Forces of the Earth come to a mature fermentation, which by some accident arriving towards its surface, as by dry weather and south winds . . . its malign effluvia or fume or vapour is drawn forth into the Aire and driven hither and thither into what place the great Guider of them and all things pleaseth." That is his explanation of the origin of the Great Plague.

The epidemic apparently began in the West and North-West of London and spread gradually across the city, " like a dark cloud," writes Defoe, " that passes over our heads which as it thickens and overcasts the air at one end clears up at the other end; so while the plague went on raging from west to east, as it went forwards east it abated in the west, by which means those parts of the town which were not seized, or who were left, and where it had spent its fury were, as it were, spared to help and assist the other. . . ." London in those days had a population of about 460,000, and in the year 1665 there were some 68,596 deaths attributed to Plague. The highest mortality in any week occurred between September 12 and 19, when there were recorded 8,297 Plague deaths. This epidemic of Plague, like those of our own time in India and elsewhere, declined gradually in malignancy. Boghurst reports that in the later cases recovery was not uncommon, that the buboes suppurated and three out of four, or three out of five, patients recovered.

With this epidemic, as with others, there was a large amount of poverty and destitution in the City. The Lord Mayor of London with the Sheriffs and Aldermen and Members of the Common Council published a resolution that " they would not quit the City themselves, but that they would be always at hand for the preserving of good order in every place, and for the doing justice on all occasions; as also for the distributing of public charity to the poor. . . ." Money was forthcoming from all parts of England for the relief of the City, and the King gave a thousand pounds a week. The arrangements made by the Lord Mayor seem to have been admirable. There was an abundant supply of food in the markets, excellent order was kept, day and night watchmen were provided to guard the infected houses, and the dead were buried quickly.

The Apothecary Boghurst gives an account of the symptoms and prognosis in plague cases. One of the worst signs, according to him, and presaging a fatal attack, was the appearance of the " Tokens " or spots, either coal black, dark bluish black, purple,

**Notable Epidemics**—(*cont*.).

clear blood-red or scarlet—in other words, petechial hæmorrhages. Continual vomiting and diarrhœa were also evil signs, and he gives a list of forty-six signs and symptoms which he considers of bad prognostic significance. Regarding the prevention of the disease he thinks, quaintly enough, that "sometimes many petty impurities keep off greater fits of sickness, and hence perhaps comes that proverb of a grunting wife, &c. Such diseases as these are commonly as good as preservatives, but I would not have anybody to trust to them. Old outward sores or ulcers, cancers, polypus, ozœna, boils, King's Evil, itch, French Pox, scabs, scaldheads, leprosy, fistula and running sores; and that such things as these should fence people against the plague is not against reason nor experience . . . witness all the common prostitutes in Lukener's Lane and Dog Yard and other places where all these unclean beasts are still alive, and scarce are dead, yet are almost all of them full of old sores, itch, scabs, running sores, pox, fistulas, ulcers and such like, so that those that made use of them had need of stomachs like ostriches, able to digest a tenpenny nail." He devotes many pages to a dissertation on the treatment and cure of plague and gives many long and complex prescriptions, rather in the fashion of the Witch's Brew. He gives recipes for an electuary, for lozenges, for posset drinks, for a poultice, a plaster, a cordial broth and for other concoctions, and he seems to have had a real belief in the value of his medicaments.

The signs which foreshow the coming of a plague are given by Boghurst as follows. Many of them, it will be noted, are signs or natural events which occurred during 1921 in this country, and it will be interesting to see if his predictions are worthy of credence to-day:—

1. Times and seasons altering their common state
2. Many changes of weather in a short time.
3. Comets, gleams of fire and fiery impressions in the air.
4. Presence of vermin, as frogs, toads, mice, flies ants, &c.
5. Death of cattle, as horses, sheep, hogs, &c.
6. Famine; also war.
7. Children in sport fancying and aping out funerals.
8. South and west winds.
9. The smallpox or spotted fever growing very rife.
10. Extraordinary ebbing and flowing of springs and rivers.
11. Much cloudy weather without rain.
12. A very dry spring, such a one as we had six months together.
13. The nights very cold and the days very hot. also a cold summer.
14. Birds, wild fowl and wild beasts leaving their accustomed places; few swallows were seen in the years 1664 and 1665.
15. Women miscarrying upon every slight occasion.
16. Fruit corrupted or altered.
17. The conditions of the stars if you will believe the astrologers.
18. The spotted fever growing rife; the measle and other epidemical diseases.
19. Generally any unusual change in the elements or creatures, whereby it seems Nature to be out of course.

The list makes formidable reading, and from it we might expect that plagues would have remained epidemic in England from that day to this!

---

### THE GREATER LONDON INQUIRY.

### How Evidence will be Taken.

THE following is an extract from a circular letter, dealing with the presentation of evidence before the Greater London Commission, which has been sent to the local authorities affected:—

The Commission have given careful consideration to the question of arranging to call witnesses in such a way as to promote the economical and expeditious conduct of the inquiry entrusted to them.

Certain authorities have inquired whether they will be separately heard in evidence by the Commission. The Commission do not wish to exclude any material evidence, but they are of opinion that a proper statement of the views of each authority concerned need not involve the presentation of separate evidence from each authority, and that the submission of separate statements, either orally or in writing, should only be made in so far as the interests of an authority are distinct from those of any other, and then only to the extent to which such distinction prevails.

The Commission earnestly hope that all authorities will accept this principle as governing the preparation of their evidence, and for the guidance of authorities in determining the practical application of the principle they have decided:—

(1) That the meetings of the Commission at which oral evidence is heard shall be open to the public and to representatives of the Press; and

(2) That the evidence of the London County Council shall be taken as soon as possible, so that any proposals made by that authority may be available for the information of other authorities concerned.

The Commission trust that this procedure will enable authorities to enter into consultation at an early date, and to formulate proposals either for the joint submission of the whole of their evidence, or for the joint submission of the major part of the evidence, supplemented by evidence on particular subjects by authorities differently grouped for the purpose, or, if requisite, by separate authorities.

The Commission have decided that persons and bodies heard in evidence before them should not in the first instance be represented by counsel.

